# Heritability and genome-wide analyses of problematic peer relationships during childhood and adolescence

**DOI:** 10.1007/s00439-014-1514-5

**Published:** 2014-12-17

**Authors:** Beate St Pourcain, C. M. A. Haworth, O. S. P. Davis, Kai Wang, Nicholas J. Timpson, David M. Evans, John P. Kemp, Angelica Ronald, Tom Price, Emma Meaburn, Susan M. Ring, Jean Golding, Hakon Hakonarson, R. Plomin, George Davey Smith

**Affiliations:** 1 MRC Integrative Epidemiology Unit (MRC IEU), University of Bristol, Oakfield House, Oakfield Grove, Bristol, BS8 2BN UK; 2 School of Oral and Dental Sciences, University of Bristol, Bristol, UK; 3 School of Experimental Psychology, University of Bristol, Bristol, UK; 4 School of Social and Community Medicine, University of Bristol, Bristol, UK; 5 Department of Psychology, University of Warwick, Coventry, UK; 6 MRC Social Genetic and Developmental Psychiatry Centre, Institute of Psychiatry, King’s College London, London, UK; 7 Department of Genetics, Evolution and Environment, Genetics Institute, UCL, London, UK; 8 Children’s Hospital of Philadelphia, Philadelphia, USA; 9 ZilkhaNeurogenetic Institute and Department of Psychiatry, University of Southern California, Los Angeles, USA; 10 Diamantina Institute, Translational Research Institute, University of Queensland, Brisbane, Australia; 11 Department of Psychological Sciences, Birkbeck, University of London, London, UK; 12 Institute for Translational Medicine and Therapeutics, University of Pennsylvania School of Medicine, Philadelphia, USA; 13 Centre for Child and Adolescent Health, University of Bristol, Bristol, UK

## Abstract

Peer behaviour plays an important role in the development of social adjustment, though little is known about its genetic architecture. We conducted a twin study combined with a genome-wide complex trait analysis (GCTA) and a genome-wide screen to characterise genetic influences on problematic peer behaviour during childhood and adolescence. This included a series of longitudinal measures (parent-reported Strengths-and-Difficulties Questionnaire) from a UK population-based birth-cohort (ALSPAC, 4–17 years), and a UK twin sample (TEDS, 4–11 years). Longitudinal twin analysis (TEDS; *N* ≤ 7,366 twin pairs) showed that peer problems in childhood are heritable (4–11 years, 0.60 < twin-*h* ^2^ ≤ 0.71) but genetically heterogeneous from age to age (4–11 years, twin-*r*_g_ = 0.30). GCTA (ALSPAC: *N* ≤ 5,608, TEDS: *N* ≤ 2,691) provided furthermore little support for the contribution of measured common genetic variants during childhood (4–12 years, 0.02 < GCTA-*h* ^2^(Meta) ≤ 0.11) though these influences become stronger in adolescence (13–17 years, 0.14 < GCTA-*h* ^2^(ALSPAC) ≤ 0.27). A subsequent cross-sectional genome-wide screen in ALSPAC (*N* ≤ 6,000) focussed on peer problems with the highest GCTA-heritability (10, 13 and 17 years, 0.0002 < GCTA-*P* ≤ 0.03). Single variant signals (*P* ≤ 10^−5^) were followed up in TEDS (*N* ≤ 2835, 9 and 11 years) and, in search for autism quantitative trait loci, explored within two autism samples (AGRE: *N*_Pedigrees_ = 793; ACC: *N*_Cases_ = 1,453/*N*_Controls_ = 7,070). There was, however, no evidence for association in TEDS and little evidence for an overlap with the autistic continuum. In summary, our findings suggest that problematic peer relationships are heritable but genetically complex and heterogeneous from age to age, with an increase in common measurable genetic variation during adolescence.

## Introduction

One of the most important developmental tasks during childhood and adolescence is the acquisition of social skills that permit the formation of successful peer relationships (Fabiano et al. 2009). Social experience during early life sets children on trajectories of positive or negative development that will continue over time and peer interaction plays an important role in the development of normal and abnormal behaviour (Fabiano et al. 2009; Fabes et al. 2011). Social acceptance, positive friendships and good social skills predict positive developmental outcomes in the long term (Fabiano et al. 2009), including educational outcomes (Risi et al. 2003). Poor social skills, lack of friendships and rejection by peers, by contrast, often precede later maladjustment (Fabiano et al. 2009), such as dropping out of school, academic difficulties, criminality, and psychopathology (Parker and Asher 1987; Ollendick et al. 1992; Schneider 2000). Despite their developmental importance, knowledge about the genetic factors influencing problematic peer relationships is scarce.

Some cross-sectional twin studies have shown that individual differences in peer-related problems are indeed heritable throughout childhood (3 years (Benish-Weisman et al. 2010): *h* ^2^ = 0.44; 12 years (Trzaskowski et al. 2013): *h* ^2^ = 0.78) though little is known about how representative these findings are within a longitudinal context including whether genetic factors are stable over time. Given variability in friendship networks during development (Gifford-Smith and Brownell 2003), it is important to investigate genetic influences within a developmental (and therefore peer) context (Ronald 2011). It is furthermore unclear whether common genetic variants contribute to the genetic architecture of peer problems and are accessible with current genome-wide designs. Recent studies reported little evidence for measurable common genetic effects influencing problematic peer behaviour during late childhood (Trzaskowski et al. 2013), based on the discordance between twin heritability and DNA-based heritability estimates using Genome-Wide Complex Trait Analysis (GCTA) (Yang et al. 2010). Investigations of other behavioural traits within population-based samples, however, showed that DNA-based heritability can increase during later adolescence (St Pourcain et al. 2014). This is supported by a meta-analysis of twin studies, which reported developmental heritability changes for multiple behavioural phenotypes. This includes, for example, a cross-time heritability increase for externalising behaviours, anxiety symptoms, depressive symptoms, intelligence quotient scores, and social attitudes from late childhood to adolescence and early adulthood (Bergen et al. 2007), and may imply that the accessibility of behavioural traits using a genome-wide association study (GWAS) design varies by age.

It is furthermore possible that some of the links between early peer problems and later maladaptive functioning are mediated through an underlying psychopathological dimension. Deficits in social interaction are, for example, a core symptom of Autism Spectrum Disorders (ASD), a severe childhood neurodevelopmental condition (American Psychiatric Association 1994), and problematic peer relationships have been related to autistic trait measures in population-based samples (Posserud et al. 2008). However, peer problems are a broad phenomenon and related to a variety of conditions, including also ADHD and Tourette syndrome (Stokes et al. 1991; Bagwell et al. 2001), and may not necessarily reflect an autistic-like phenotype. If, however, peer problems scores do represent a broader autistic trait, it could be assumed that there might be autism quantitative trait loci (QTL) affecting both subtle variation in peer relationships and risk of autism, as recent twin studies found evidence for aetiological similarity between ASD and autistic traits, including similar heritability estimates at both ends of the autistic continuum (Robinson et al. 2011; Lundström et al. 2012).

Using a series of longitudinal measures spanning early childhood till later adolescence (4–17 years) from a representative population sample in the UK (Avon Longitudinal Study of Parents and Children, ALSPAC), and a representative UK twin sample (Twins Early Development Study, TEDS), this study aimed to investigate the genetic architecture of problematic peer behaviour from a developmental perspective. Specifically, we studied evidence for additive genetic effects using a longitudinal twin design and performed multiple cross-sectional GCTA-heritability analyses. This was followed by GWAS focussing on the phenotypic measures with the highest evidence for measurable common genetic influences. We finally explored the strongest population-based single genetic association signals also in two autism samples.

Here, we report evidence for the contribution of additive genetic influences to problematic peer relationships during development. These genetic factors are genetically heterogeneous and show an increase in common measurable genetic variation during adolescence. We found, however, no evidence for single SNP association at the genome-wide significance level at any age, and little support for an overlap with the autistic continuum.

## Materials and methods

### General population samples

GCTA and subsequent genome-wide analysis were conducted in children from ALSPAC, a UK population-based longitudinal pregnancy-ascertained birth-cohort (estimated birth date: April 1991–December 1992) (Boyd et al. 2013; Fraser et al. 2013), which is representative of the general population (~96 % White mothers). The initial cohort included 14,541 pregnancies and additional children eligible using the original enrolment definition (i.e. based on the same delivery dates) were recruited up to the age of 18 years, increasing the total number of pregnancies to 15,247. Information on children is available from questionnaires, clinical assessments, linkage to health and administrative records as well as biological samples including genetic and epigenetic information. Ethical approval was obtained from the ALSPAC Law-and-Ethics Committee (IRB00003312) and the Local Research Ethics Committees. The study website contains details of all available data (http://www.bris.ac.uk/alspac/researchers/data-access/data-dictionary).

Further GCTA, twin analyses, and a follow-up study of selected signals from the genome-wide screen in ALSPAC were carried out in TEDS, a large longitudinal sample of twins born in England and Wales between 1994 and 1996 (Haworth et al. 2013). The collected measures focus on cognitive and behavioural development, including difficulties in the context of normal development (www.teds.ac.uk). TEDS began when multiple births were identified from birth records and the families were invited to take part in the study; 16,810 pairs of twins were originally enrolled in TEDS. More than 10,000 of these twin pairs remain enrolled in the study to date. DNA has been collected for more than 7,000 pairs, and genome-wide genotyping data for two million DNA markers are available for around 3,500 individuals. Information is available on the twins using a combination of parent, teacher, and child rated questionnaire measures, home visits, linkage of records and online tests of cognition and behaviour. The TEDS families have taken part in studies roughly once every 2 years since the twins were 18 months of age. Ethical approval for each stage of TEDS has been obtained from the Institute of Psychiatry Ethics Committee, and informed consent was collected from the parents for each assessment. Further details about the composition and representativeness of the sample, and an overview of the measures collected are available elsewhere (Haworth et al. 2013).

### Measurement of peer problems

Problematic peer relationships in ALSPAC and TEDS children were measured with the parent-completed 5-item peer problems subscale of the Strengths-and-Difficulties questionnaire (SDQ, (Goodman 1997)). The SDQ is a widely used (http://www.sdqinfo.org/py/sdqinfo/f0.py), short behavioural screening instrument applicable to children and adolescents ranging from 4 to 16 years (Goodman 1997). The SDQ has been developed as a screening instrument to predict several childhood developmental conditions (Goodman et al. 2003), the reliability of the SDQ peer problem scale is sufficient (internal consistency as measured by Cronbach’s *α* = 0.57) (Goodman 2001). The validity of the SDQ has been assessed by how strongly the subscales are associated with the presence of psychiatric disorders (Goodman 2001), and high SDQ scores have been associated with a substantial increase in psychiatric risk. For the peer problem subscale, there was a prevalence of a DSM-IV diagnosis of 6.4 % in the low-risk group and 31.3 % in the high-risk group (i.e. in the extreme 10 % of the population) (Goodman 2001). Different SDQ scoring profiles (including items of the peer problem scale) have been shown in patients with different clinical diagnoses, including, for example, elevated levels of peer problems and emotional difficulties, and fewer prosocial behaviours in children with ASD compared to children with ADHD (Iizuka et al. 2010).

The peer problem subscale includes the items: (I) “Rather solitary, tends to play alone”; (II) “Has at least one good friend”; (III) “Generally liked by other children”; (IV) “Picked on or bullied by other children”; and (V) “Gets on better with adults than with other children”. Each item was rated as “not true” (0), “somewhat true” (1) or “certainly true” (2) and items (II) and (III) were reverse-coded (Goodman 1997). All items were eventually summed to give a final peer problem score (score-range 0–10) with higher scores reflecting more peer-related problems. Quantitative mother-reported SDQ peer problem scores in ALSPAC children and adolescents were measured at 4, 7, 8, 10, 12, 13 and 17 years of age, and in TEDS participants parent-reported scores are available at 4, 7, 9 and 11 years (Table 1). Correlations between the scales at different ages showed modest to moderate stability in both ALSPAC (Spearman’s rho (*ρ*): 0.22 < *ρ* < 0.57; Supplementary Table S1) and TEDS (Spearman’s rho: 0.27 < *ρ *< 0.49; Supplementary Table S2). As expected, assessments closer in age were more strongly correlated than those that spanned the entire developmental period (Supplementary Tables S1 and S2).

**Table 1:** ALSPAC and TEDS sample characteristics

Age in years							
	4	7	8	9–10	11–12	13	17
Twin analysis							
*TEDS*							
All Mean (SD)^a^	1.45 (1.47)	0.97 (1.39)	–	1.06 (1.52)	1.07 (1.49)	–	–
All N	7,366	7,205	–	3,258	5,600	–	–
MZ Mean (SD)^a^	1.31 (1.37)	0.83 (1.28)	–	0.92 (1.43)	1.00 (1.42)	–	–
MZ N	2,534	2,596	–	1,206	2,040	–	–
DZ Mean (SD)^a^	1.53 (1.51)	1.05 (1.44)	–	1.14 (1.57)	1.11 (1.53)	–	–
DZ N	4,832	4,609	–	2,052	3,560	–	–
Genetic association analysis/GCTA							
*ALSPAC*							
Mean (SD)	1.49 (1.51)	1.02 (1.40)	1.28 (1.53)	1.11 (1.50)	1.1 (1.56)	1.19 (1.62)	1.11 (1.51)
Age in years (SD)	3.99 (0.13)	6.79 (0.11)	8.17 (0.14)	9.65 (0.12)	11.72 (0.13)	13.16 (0.18)	16.84 (0.36)
Males (%)	51.42	50.97	50.68	50.50	49.71	49.65	48.36
*N* ^b^	6,000	5,690	5,259	5,747	5,337	5,134	4,214
*TEDS*							
Mean(SD)	1.40 (1.45)	0.92 (1.39)	–	1.04 (1.54)	1.07 (1.5)	–	–
Age in years (SD)	4.03 (0.12)	7.05 (0.25)	–	9.00 (0.28)	11.26 (0.69)	–	–
Males (%)	45.51	45.40	–	45.45	45.94	–	–
*N* ^b^	2,628	2,837	–	1,507	2,708	–	–

*MZ* monozygotic twins (including incomplete pairs), *DZ* dizygotic twins (male, female, opposite sex; including incomplete pairs), *GCTA* genome-wide complex trait analysis

^a^Based on one randomly selected member of each twin pair

^ b^Individuals with genotypic and phenotypic data

### Twin analysis

Twin analyses were used to estimate the relative contribution of genetic and environmental influences to individual differences in quantitative peer problem scores. Twin intraclass correlations were calculated (Shrout and Fleiss 1979), providing an initial indication of the relative contributions of additive genetic (A), shared environmental (C), and non-shared environmental (E) factors. Additive genetic influence, also commonly known as heritability, is estimated as twice the difference between the identical and fraternal twin correlations. In twin analysis, additive genetic influences (A) include all additive genetic effects both from rare and common variants, whereas GCTA provides a lower limit estimate of heritability (A) as genetic influences due to causal variants that are not highly correlated with the common SNPs on genotyping arrays, including rare variants, are not captured (Yang et al. 2010; Plomin et al. 2013). The contribution of the shared environment, making members of a family similar, is estimated as the difference between the identical twin correlation and heritability. Although the influence of shared environment (C) was non-significant (see “Results”), twin analysis was carried out using the full ACE model to allow for comparison with GCTA estimates. Removing the influence of shared environment (C) from the analysis model could have inflated the effect of additive influences (A) and thus affected the comparison with additive influences (A) as provided by GCTA (Trzaskowski et al. 2013). Non-shared environments, i.e. environments specific to individuals, were estimated by the difference between the identical twin correlation and 1 because they are the only source of variance making identical twins different. Estimates of the non-shared environment also include measurement error. Maximum likelihood structural equation model-fitting analyses were carried out to allow for more complex analyses of the relative contribution of A, C and E (Rijsdijk and Sham 2002) and standard twin model-fitting analyses were conducted using the Mx software (Neale et al. 2006). All twin analyses were carried out using untransformed peer problem scores at 4, 7, 9 and 11 years of age that were ascertained in up to 7366 TEDS twin pairs. Detailed information on the analysed twin sample can be found in Table 1.

Multivariate (longitudinal) twin analyses were used to go beyond estimating the cross-sectional importance of genetic and environmental factors and to consider the degree to which genes and environments important at one age are also important at later ages (Neale et al. 2006). We used a standard Cholesky decomposition, converted to the mathematically equivalent correlated factors solution, to estimate the degree of genetic and environmental overlap between our longitudinal measures. In univariate twin analyses, we break down the phenotypic variance into genetic and environmental sources. The exact same logic is used in multivariate analyses to decompose the covariance between traits (or, as in the present case, between the ‘same’ trait at different ages) into genetic and environmental sources. The main outcome measures from these twin analyses are indices of genetic, shared and non-shared environmental correlations between our measured peer problems scales at ages 4, 7, 9 and 11. These correlations can range from −1 to +1, and the point estimates are independent of the magnitude of the genetic and environmental influence on each trait. Therefore, it is possible to have, for example, a high shared environmental correlation between ages even when the shared environmental influence at each age is small in magnitude, although the confidence intervals for correlations based on small proportions of variance are typically large. Such a result would mean that of the limited shared environmental variance present at each age, most of this variance also influences the later age. It is, therefore, important to interpret genetic and environmental correlations within the context of the magnitude of the cross-sectional magnitude of the A, C and E factors.

### Genotyping and imputation

ALSPAC children were genotyped using the Illumina HumanHap550 quad-chip array. Genotypes were cleaned as previously described using standard quality control methods (Paternoster et al. 2012). In summary, single nucleotide polymorphisms (SNPs) with a minor allele frequency (MAF) <1 %, a call rate <95 % or evidence for violations of Hardy–Weinberg equilibrium (*P* < 5.0 × 10^−7^) were excluded. Individual participant samples were removed on the basis of sex mismatches, minimal or excessive heterozygosity, disproportionate levels of individual missingness, cryptic relatedness, insufficient sample replication and non-European ancestry. Using 464,311 directly genotyped SNPs, genotypes for 8,365 independent individuals (irrespective of available phenotypic data) were imputed to HapMapCEU (Utah residents with Northern and Western European ancestry from the Centre d’Etude du PolymorphismeHumain collection) individuals (Rel 22) using MACH (Li et al. 2010).

TEDS children were genotyped at the Affymetrix service laboratory using the Affymetrix GeneChip 6.0 and data were cleaned as previously described (Davis et al. 2014). In brief, 3,665 DNA samples from unrelated children (one member of a twin pair) were successfully genotyped. Individual samples were excluded because of low call rate or heterozygosity outliers, intensity outliers, ancestry outliers, relatedness/duplicates, gender mismatches or low concordance (<90 % after re-genotyping on a panel of 30 SNPs using Sequenom). SNPs were excluded based on minor allele frequency (MAF < 1 %) and Hardy–Weinberg (*P* < 10^−6^). SNPs with greater probability of a null call were down-weighted in the analysis, thresholding at 0.9. Imputation was carried out using the IMPUTE2 software on clean genotype data by a two-stage approach with both a haploid reference panel (HapMap2 and HapMap3 SNP data on the 120 unrelated HapMap CEU trios (Rel 22)) and a diploid reference panel (5,175 WTCCC2 controls) as previously described (Davis et al. 2014).

To increase the effective sample size and power of our analysis, we used imputed genotype data for the genetic association analysis. This allows the exchange and combination of genotype data in a uniformly exchangeable format (de Bakker et al. 2008), even when genotypes are collected using different genotyping platforms.

In addition, ancestry-specific principal components were calculated with Eigenstrat (Price et al. 2006) within each cohort (using raw genotypes), to correct for subtle differences in population structure.

All reported LD-measures are based on HapMap CEU (Rel22).

### Estimation of GCTA-heritability

Using GCTA (Yang et al. 2010), we estimated the proportion of additive phenotypic variation explained by all genotyped SNPs together, both in ALSPAC (at 4, 7, 8, 10, 12, 13 and 17 years of age) and in TEDS (at 4, 7, 9, and 11 years of age). Pertinent to this study, GCTA was carried out using untransformed peer problem scores in each cohort and the most likely imputed as well as direct genotypes from autosomal SNPs (ALSPAC: *N*_SNPs_ = 2,449,665, MAF ≥ 0.01, imputation accuracy MACH-*R* ^2^ > 0.3; TEDS: *N*_SNPs_ = 1,588,650 (MAF ≥ 0.01 and INFO > 0.7 score). For sensitivity analysis, GCTA was also performed using peer problem scores adjusted for age, sex and the two most significant principal components, in addition to adjusted and subsequently rank-transformed scores. GCTA estimates from ALSPAC and TEDS were combined using fixed-effects inverse-variance meta-analysis, and evidence for overall heterogeneity was tested using Cochran’s* Q*-test.

We also used bivariate GCTA (Lee et al. 2012) to estimate the extent to which the same genes contribute to the observed phenotypic correlation between two variables. These estimations are based on the genetic covariance between untransformed peer problem measures at different ages, which is due to common measured genetic variation.

### Genetic association analysis

Selecting peer problem scores with the highest GCTA-heritability during development (10, 13 and 17 years of age), we conducted three single time-point GWASs on ~2.45 million (*N* = 2,449,665) common imputed and genotyped SNPs (MAF ≥ 0.01, imputation accuracy MACH-*R* ^2^ > 0.3) within ALSPAC. Association analyses were performed using a quasi-Poisson regression model, which can accommodate overdispersion (Faraway 2006) (*R ʻ*stats’ library). Specifically, counts of peer problems were regressed on allele dosage as well as age, sex and the two most significant ancestry-informative principal components (to correct for subtle differences in population structure (Price et al. 2006)). Regression estimates (*β*) thus represent changes in log-counts of peer problems per effect allele, based on SNP dosage scores. All single time-point findings were subjected to genomic control (GC)-correction (Devlin and Roeder 1999). Follow-up analyses were carried out in TEDS using a similar quasi-Poisson regression framework as described for ALSPAC including two ancestry-informative principal components.

### Exploratory analysis of population-based association signals in two autism samples

Population-based signals were also investigated in the Autism Genetic Resource Exchange (AGRE) pedigrees and the Autism Case–Control (ACC) cohort in an exploratory search for autism QTL. Within the AGRE pedigrees, there are three diagnostic categories based on the Autism Diagnostic Interview–Revised (ADI–R) (Lord et al. 1994): Autism, Broad Spectrum or Not Quite Autism. All of them were utilised to define ‘cases’ in this study, and have been previously described in detail (Wang et al. 2009). 4,444 unique AGRE individuals from 943 families were genotyped on the Illumina HumanHap550 K BeadChip (Wang et al. 2009). Cleaned genome-wide data (Wang et al. 2009) were obtained from Autism Speaks (data set prepared by JK Lowe). Additional data cleaning steps of this multiethnic sample have been described in detail in previous publications (St Pourcain et al. 2014) including the removal of SNPs (>10 % missingness, violations of Hardy–Weinberg equilibrium (*P* < 0.001) and MAF <1 %) as well as the exclusion of individuals (>10 Mendelian errors, monozygotic twins, sample duplicates, individuals with >10 % missing data, individuals with known chromosomal abnormalities including Trisomy 21 and Fragile X syndrome, individuals of non-European ancestry). The final data set included 3,299 individuals (793 pedigrees) and 513,312 SNPs. Genotypes were imputed to HapMap CEU (release 22) using MaCH, excluding all imputed genotypes with a per-genotype posterior probability <0.9. Selected population-based signals were investigated with FBAT, a family-based association test (Lange and Laird 2002), using the most likely genotype call and an empirical variance for the test statistic (to account for linkage within pedigrees).

The ACC cohort includes 1,453 patients with either a positive ADI/ADI–R score or an Autism Diagnostic Observation Schedule (Lord et al. 2000) diagnosis or both, as well as 7,070 controls without a history of ASD. All individuals were genotyped on the Illumina HumanHap550 K BeadChip. The data cleaning was largely similar to the cleaning of the AGRE sample (see above) and has been described previously (Wang et al. 2009). The final clean data set included 1,204 ASD cases and 6,491 controls of European ancestry, as well as 480,530 SNPs (Wang et al. 2009). Genotype imputation was performed to HapMap CEU (release 22) using MaCH as previously reported (Wang et al. 2009). Genetic association for selected follow-up SNPs was analysed using SNPTEST by converting MaCH imputation files into SNPTEST input formats.

Genetic association analysis was conducted using de-identified genetic data. Ethical approval for the analysis of the AGRE and ACC samples was obtained through the IRB Protocol 10-007590 from the Children’s Hospital of Philadelphia.

### Longitudinal modelling of DNA signals

All population-based signals in ALSPAC with tentative support for autism QTL were furthermore modelled longitudinally. For this, we used a mixed Poisson regression framework (R:‘lme4’ library), where overdispersion can be modelled through the random error part (Gelman and Hill 2007). Models included random intercept and slope, and SNP effects (i.e. allele dosages) were adjusted for sex, age, age^2^ and two ancestry-sensitive principal components. In addition, we modelled age-specific SNP effects using SNP × age and SNP × age^2^ interaction terms, and selected the best-fitting model based on likelihood-ratio tests. Thus, for each SNP, the final longitudinal model could include none, one (SNP × age) or two (SNP × age and SNP × age^2^) interaction effects. In the presence of SNP–age interaction effects, we modelled the SNP effect at different ages spanning early childhood (4 years) and later adolescence (17 years), by centering age at the respective age. We considered a SNP signal of 5 × 10^−8^ at any age (including combined effects from main and interaction effects) within the longitudinal modelling framework as genome-wide significant.

## Results

### Heritability analyses

**Fig. 1:**
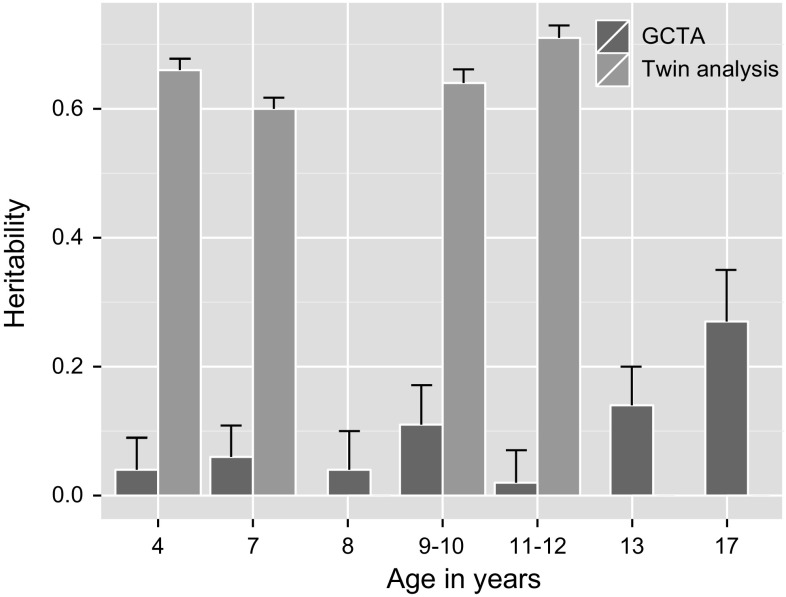
Twin and Genome-wide Complex Trait (GCTA) heritability estimates for problematic peer relationships during childhood and adolescence. GCTA-heritability was derived from meta-analysis (ALSPAC + TEDS: 4, 7, 9–10 and 11–12 years of age) or single sample estimates (ALSPAC: 8, 13 and 17 years of age). *Error bars* indicate standard errors (SE). *N* = 3,258–7,366 twin pairs for twin analysis (TEDS), and *N* = 4,007–8,219 unrelated individuals for GCTA (ALSPAC, TEDS). Detailed information on twin and GCTA estimates is provided in Supplementary Tables S3 and S4, respectively

**Fig. 2:**
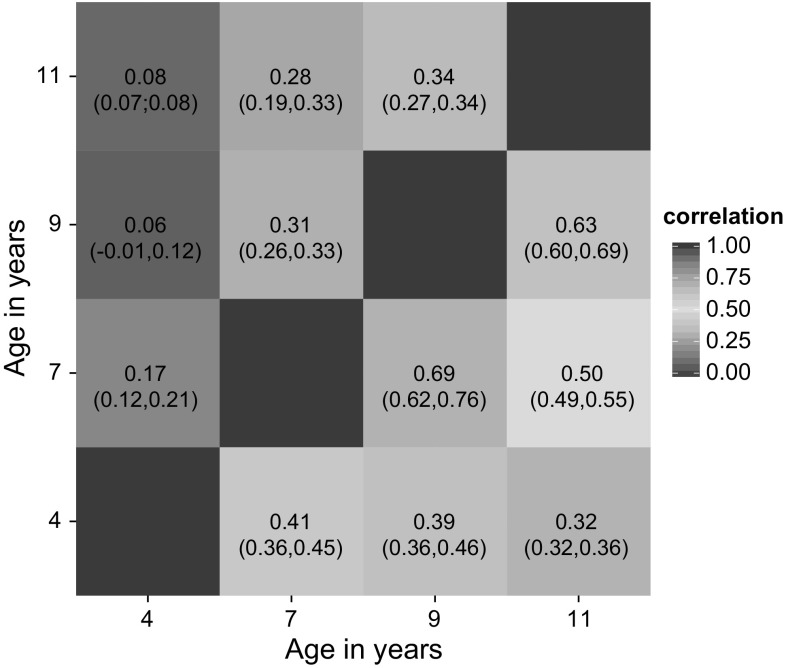
Genetic and specific environmental correlations between problematic peer relationships during development. Estimates were derived from longitudinal twin analysis (ACE model). *Lower triangle* genetic correlations (*r*_g_); *upper triangle* specific environmental correlations (*r*_e_). All correlations are given with their 95 % confidence intervals (colour figure online)

Peer problems during childhood and adolescence are interrelated, both within children of the general population (ALSPAC, 4–17 years of age, Supplementary Table S1) and children from a national twin sample (TEDS, 4–11 years of age, Supplementary Table S2). Longitudinal twin analysis in TEDS (Fig. 1, Supplementary Table S3) indicates that problematic peer behaviour throughout early to late childhood is highly heritable (4–11 years, 0.60 < twin-*h* ^2^ ≤ 0.71), with negligible shared environmental effects (0.02 < *c* ^2^ ≤ 0.09) and moderate non-shared/residual influences (4–11 years, 0.27 < twin-*e* ^2^ ≤ 0.38). Twin modelling furthermore provided evidence for considerable genetic heterogeneity during development with a genetic correlation of just 0.32 between ages 4 and 11 (Fig. 2, Supplementary Table S3). The degree of genetic overlap does appear to increase with age (*r*_g_ = 0.63 between ages 9 and 11, for example), suggesting increased stability in genetic influences into early adolescence (Supplementary Table S3). Non-shared environmental overlap (Fig. 2, Supplementary Table S3) is lower than genetic overlap, indicating that the environmental influences important for peer problems are largely specific to each developmental stage, or peer context. Estimates of shared environmental correlations are also provided in Supplementary Table S3.

GCTA based on samples of independent children from ALSPAC and TEDS (including a subsample of children used for twin analysis) revealed that measured common additive genetic effects explained only a modest amount of variance during early to late childhood (4–12 years, 0.02 < GCTA-*h* ^2^(Meta) ≤ 0.11, *P*_min_ = 0.04; Fig. 1; Supplementary Table S4). However, common genetic influences appear to become stronger during adolescence (13–17 years, 0.14 < GCTA-*h* ^2^(ALSPAC) ≤ 0.27, 0.0002 < *P* ≤ 0.01; Fig. 1; Supplementary Table S4).These findings are independent of adjustment for age, gender and principal components (Supplementary Table S5) and phenotype transformation (Supplementary Table S6). Bivariate GCTA estimations were carried out for ALSPAC peer problem scores with the highest GCTA-heritability in adolescence (i.e. 13 and 17 years) and there was at least suggestive evidence for genetic correlations from age to age (GCTA-*r*_g_ = 0.53 (SE = 0.24), *P* = 0.05), irrespective of phenotype adjustment (see above, Supplementary Table S5) and phenotype transformation (Supplementary Table S6). GCTA-based genetic correlations for peer problem measures during childhood are not reported because of estimation problems due to low GCTA-*h* ^2^.

### Genome-wide single variant analyses

Findings from twin analysis and GCTA, showing increased magnitude and stability for additive genetic influences with age, informed the design of a two-stage GWAS, as GCTA-heritability is one of the key factors that influence study power. Based on the analysis of untransformed scores (Fig. 1; Supplementary Table S4), we selected ALSPAC phenotypes with the highest GCTA-heritability (i.e. at 10, 13 and 17 years, 0.11 < GCTA-*h* ^2^(ALSPAC) ≤ 0.27, 0.0002 < GCTA-*P* ≤ 0.03). During the discovery stage, three genome-wide screens were carried out within children from the general population (ALSPAC), thus allowing for genetic heterogeneity during development through a cross-sectional design. At these ages, association signals showed no genome-wide significant deviation from the null hypothesis and there was little evidence for population stratification (1.023 < λ_GC_ ≤ 1.034; Quantile–Quantile plots are shown in Supplementary Figure S1). The strongest genetic associations with problematic peer relationships were identified on chromosome 12p12.1 within *ST8SIA1* (10 years: rs722248 GC-corrected *P* = 2.1 × 10^−6^), on chromosome 10p15.1 near *KLF6* (13 years: rs7898258 GC-corrected *P* = 2.4 × 10^−7^) and on chromosome 2p22.2 near *CRIM1* (17 years: rs3770890 GC-corrected *P* = 5.3 × 10^−7^). None of the signals showed consistent evidence for association (*P* < 10^−5^) throughout development. However, independent SNPs within *CRIM1* (Linkage disequilibrium (LD) *r* ^2^ < 0.3) were associated with problematic peer relationships during different stages of development (see Table 2).

**Table 2:** Single time-point genome-wide association analysis in ALSPAC (genomic-control corrected *P* < 1 × 10^−5^)

Age in years											
GWAS	SNP	E/A	EAF	Chr	Gene	10 (*N* = 5,747)		13 (*N* = 5,134)		17 (*N* = 4,214)	
						Beta (SE)^a^	*P* ^a^	Beta (SE)^a^	*P* ^a^	Beta (SE)^a^	*P* ^a^
Age 10	rs6699546	A/G	0.75	1q25.1	*TNR*	−0.13 (0.03)	7.3 × 10^−6^	−0.08 (0.03)	0.013	−0.12 (0.03)	6.3 × 10^−4^
	rs11903722	A/G	0.54	2p25.1	*AX746649*	0.12 (0.03)	6.2 × 10^−6^	0.02 (0.03)	0.47	0.02 (0.03)	0.58
	rs3770951	T/C	0.18	2p22.2	*CRIM1*	0.15 (0.03)	2.1 × 10^−6^	0.11 (0.03)	6.5 × 10^−4^	0.13 (0.04)	7.8x10^−4^
	rs9650197	T/C	0.46	8q12.1	*CA8*	0.12 (0.03)	2.6 × 10^−6^	0.05 (0.03)	0.093	0.03 (0.03)	0.32
	rs722248	A/G	0.24	12p12.1	*ST8SIA1*	−0.15 (0.03)	2.1 × 10^−6^	−0.08 (0.03)	0.016	0.01 (0.04)	0.78
	rs7166089	T/C	0.70	15q26.3	*PCSK6*	−0.12 (0.03)	8.0 × 10^−6^	−0.07 (0.03)	0.014	−0.04 (0.03)	0.27
Age 13	rs7873232	A/G	0.35	9p24.2	*GLIS3*	0.11 (0.03)	8.4 × 10^−5^	0.13 (0.03)	7.3 × 10^−6^	0.08 (0.03)	0.013
	rs7898258	A/C	0.67	10p15.1	*KLF6*	0.06 (0.03)	0.031	0.16 (0.03)	2.4 × 10^−7^	>0.001 (0.03)	0.96
	rs11019786	A/T	0.05	11q14.3	*FAT3*	0.11 (0.05)	0.041	0.24 (0.05)	5.2 × 10^−6^	0.02 (0.07)	0.73
	rs9543667	A/G	0.70	13q22.1	*BC043259*	0.04 (0.03)	0.14	0.14 (0.03)	7.6 × 10^−6^	0.001 (0.03)	0.98
	rs10775373	A/T	0.65	17p12	*RICH2*	−0.10 (0.03)	2.0 × 10^−4^	−0.12 (0.03)	8.6 × 10^−6^	−0.08 (0.03)	0.013
	rs4797686	T/G	0.03	18p11.21	*SLMO1*	0.15 (0.08)	0.063	0.36 (0.08)	4.8 × 10^−6^	0.15(0.10)	0.12
	rs6565811	A/C	0.21	18q23	*BC037384*	0.08 (0.03)	0.0088	0.14 (0.03)	4.7 × 10^−6^	0.04 (0.04)	0.23
	rs533794	A/C	0.13	22q12.1	*CR936633*	0.07 (0.04)	0.085	0.17 (0.04)	6.3 × 10^−6^	0.09 (0.04)	0.037
Age 17	rs3770890	T/G	0.97	2p22.2	*CRIM1*	−0.32 (0.07)	8.8 × 10^−6^	−0.26 (0.08)	7.0 × 10^−4^	−0.41 (0.08)	5.3 × 10^−7^
	rs17038966	A/G	0.06	4q25	*AK094992*	0.07 (0.05)	0.19	0.15 (0.05)	0.003	0.25 (0.06)	5.9 × 10^−6^
	rs6451614	A/G	0.17	5p13.1	*GHR*	−0.06 (0.04)	0.12	−0.15 (0.04)	2.3 × 10^−4^	−0.20 (0.05)	9.6 × 10^−6^
	rs6940109	T/C	0.21	6p25.2	*C6orf145*	−0.004 (0.03)	0.89	0.06 (0.03)	0.060	0.16 (0.04)	4.2 × 10^−6^
	rs6947368	A/G	0.05	7p21.3	*COL28A1*	0.02 (0.06)	0.80	0.08 (0.07)	0.21	0.29 (0.07)	7.2 × 10^−6^
	rs2007127	A/G	0.82	7q31.33	*BC031318*	−0.09 (0.03)	0.0038	−0.12 (0.03)	4.3 × 10^−4^	−0.17 (0.04)	3.5 × 10^−6^
	rs1370194	T/C	0.35	18q12.3	–	0.05 (0.03)	0.051	0.11 (0.03)	4.8 × 10^−5^	0.14 (0.03)	5.4 × 10^−6^
	rs12974813	T/C	0.21	19q13.42	*HSPBP1*	0.01 (0.03)	0.70	0.05 (0.03)	0.13	0.17 (0.04)	3.4 × 10^−6^

Results are presented for the most significant signals from independent loci within a linkage disequilibrium (LD) window (LD *r* ^2^ = 0.3, ±500 kb). Regression estimates were obtained using quasi-Poisson regression. All signals are uncorrected for multiple testing

E effect allele, A alternative allele, EAF effect allele frequency, Gene nearest gene within ±500 kb, Selection single time-point GWAS

^a^Genomic-control corrected

During the second stage of the genome-wide screen, a follow-up of independent association signals (LD-based clumping (Purcell et al. 2007): *r* ^2^ = 0.3, ±500 kb; GC-corrected cross-sectional *P* < 10^−5^ at 10, 13 or 17 years of age) from the discovery stage was carried out in a sample of unrelated children from TEDS with peer problem measures at 9 and 11 years of age (as parent-rated 5-item SDQ peer problem scores at later ages are not available). This follow-up analysis in TEDS (Supplementary Table S7) provided no evidence for association, assuming the same direction of genetic effect as observed in the discovery cohort.

**Fig. 3:**
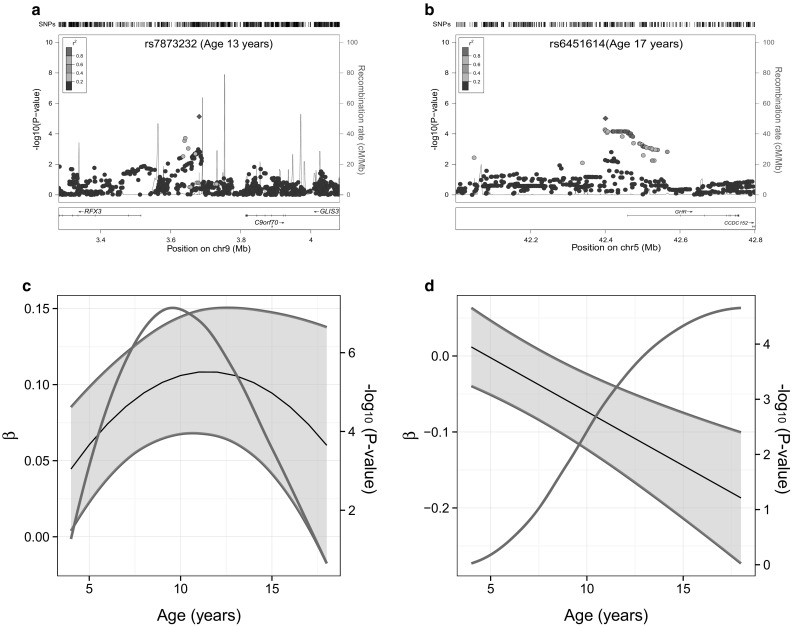
Peer problem association signals in ALSPAC. Regional association plots for rs7873232 (**a**) and rs6451614 (**b**) based on cross-sectional GWAS in ALSPAC at 13 and 17 years, respectively. The genomic position is shown in mega bases (Build 36). Recombination rates are based on HapMap CEU (Rel22) and the LD (*r* ^2^) between the lead variant and surrounding SNPs is indicated by the colour code. Developmental changes in the genetic association at rs7873232 (**c**) and rs6451614 (**d**) in ALSPAC. Longitudinal modelling was carried out with a mixed Poisson model using all available data between 4 and 17 years of age. Genetic effects (β) and their 95 % confidence intervals (*black* and *blue lines*) represent the change in log-counts of peer problems per effect allele (rs7873232_A and rs6451614_A) at different stages during development and are shown together with the strength of the genetic association (−log_10_ *P* value, *red line*). Genetic effects are based on linear combinations of SNP, SNP × age and/or SNP × age^2^ interaction effects, and longitudinal model parameters are described in Supplementary Table S9 (colour figure online)

An exploratory analysis of population-based signals in two ASD samples (Supplementary Table S8) showed, however, that two common population-based signals, rs7873232 at 9p24.2 and rs6451614 at 5p13.1 (Table 2), increased risk for autism in the AGRE sample (assuming the same direction of effect) though no such association was observed in the ACC sample (Table 3). rs7873232 resides ~170 kb 5′ of *RFX3* and ~130 kb *3′ of GLIS3* and rs6451614 is located ~60 kb 5′ of *GHR* (see Fig. 3a, b, respectively). Population-based genetic influences at both rs7873232 and rs6451614, as observed in the ALSPAC sample are, however, highly variable. Longitudinal modelling showed that variation at rs7873232 exerted a U-shaped genetic effect, which peaks during late childhood (Fig. 3c; Supplementary Table S9), while the genetic effect at rs6451614 increased linearly during development (as a negative effect) and was strongest during late adolescence (Fig. 3d; Supplementary Table S9). None of these population-based signals reached genome-wide significance at any stage during development when modelled longitudinally within ALSPAC.

**Table 3:** Single time-point ALSPAC signals (13 and 17 years) in AGRE and ACC

ALSPAC GWAS	SNP	E/A	Chr	Gene	AGRE			ACC				
					EAF	Z	*P*	Proxy SNP (*r* ^2^)	Proxy E/A	EAF^a^	OR (95 % CI)	*P*
Age 13	rs7873232	A/G	9p24.2	*GLIS3*	0.35	2.96	0.0030	–	A/G	0.35	1.00 (0.91;1.09)	0.99
Age17	rs6451614	A/G	5p13.1	*GHR*	0.18	−3.44	0.00059	rs1858136 (0.88)	C/G	0.20	1.02 (0.92;1.14)	0.69

Follow-up of signals from single time-point GWAS in ALSPAC was conducted with family-based association analysis (FBAT) in AGRE using the most likely genotypes; Within the ACC, case–control association analysis was conducted using SNPTEST; Only signals which are consistent with an autism quantitative trait locus are shown (see Supplementary Table S8 for all analyses); All SNPs had sufficient imputation quality (AGRE: 0.89 < *R* ^2^ ≤ 1 (MACH); ACC: 0.97 < PROPERINFO ≤ 1 (SNPTEST))

*AGRE* autism genetic research exchange (AGRE) sample (793 ASD pedigrees), *ACC* autism case–control cohort (1204 ASD subjects, 6491 control subjects, OR is given for the effect allele), *E* effect allele, *A* alternative allele, *EAF* effect allele frequency, *95* *%-CI* 95 % confidence interval, *r* ^2^ linkage disequilibrium coefficient; *Gene* nearest gene within ±500 kb

^a^Within ASD subjects

## Discussion

This study involved a twin and molecular genetic analysis of problematic peer behaviour during childhood and adolescence.

Twin analysis estimated that approximately 60–70 % of the phenotypic variance in problematic peer behaviour is explained by genetic differences, with most of the remaining variance explained by non-shared environmental influences. The observed heritability for problematic peer behaviour remains consistently high during development and supports previous cross-sectional findings in early (Benish-Weisman et al. 2010) and late childhood (Trzaskowski et al. 2013). This suggests that genetic influences play an important role in the development of social skills and how well children eventually integrate in social networks, which in turn will affect their social and behavioural outcomes in later life. The modest genetic stability of these traits during childhood implies, however, that the underlying genetic architecture is likely to be complex and variable during development.

Consistent with previous GCTA findings for a large number of behavioural and social traits (Trzaskowski et al. 2013; St Pourcain et al. 2014) including reports on peer problems in TEDS at 12 years (Trzaskowski et al. 2013), we observed a lack of measurable common genetic effects during late childhood. The increase in GCTA-heritability in adolescence, with ~30 % of the phenotypic variance explained by age 17, mirrored GCTA findings for social-communication traits during the same developmental stage (St Pourcain et al. 2014) suggesting that this rise in GCTA-heritability during later adolescence might be generic to behavioural traits.

Variation in GCTA-heritability for problematic peer behaviour during development may be the consequence of many underlying factors including complex alterations in the genetic architecture, especially around puberty, or changes in the interplay between genes and environments as children move between peers groups. For example, low estimates in GCTA-heritability may reflect non-additive effects, such as gene x environment (G × E) interactions. So far, we found no indication for non-additive genetic effects in our twin analyses, which would be suggested if the non-identical twin correlations are less than half the identical twin correlations. The twin method, however, is not a very powerful method for separating non-additive from additive genetic effects (Rijsdijk and Sham 2002), so we cannot rule out the possibility that problems with peer relations are affected by non-additive genetic influences. In addition, exploration of G × E effects in the twin model requires the inclusion of measured indices of environmental exposure which are not available in the TEDS sample. Nevertheless, we speculate that it is possible that developmental stages in childhood, especially those overlapping with profound social and biological changes such as puberty, may involve increased interactions between genetic and environmental effects compared to developmental periods in later adolescence, which are characterised by more uniform physical, mental and social maturation.

Alternatively, peer problems may depend on pubertal timing and relate to disparities between chronological age, social age and biological maturation (e.g. “early maturation” hypothesis (Peterson and Taylor 1980)). While there is virtually no discrepancy in biological maturation between monozygotic twins, and the developmental status in dizygotic twins is likely to be coupled due to family relationship, the highest variability in biological development during puberty, with respect to a given chronological age, will be among independent children, irrespective of whether they were drawn from a twin sample or a birth cohort. Within independent samples, therefore, any relationship between pubertal status and peer problems might have been masked, thus downward biasing the observed GCTA-heritabilities.

It is also possible, that changes in GCTA-heritability during development may reflect changes in the genetic architecture over time, due to variation in phenotype composition. For example, there are changes in the understanding of friendship and peer interaction during development (Berndt 2002). This involves some aspects of friendships, such as intimacy (‘best friends tell each other everything’) and loyalty (‘best friends stick up for each other’), which are only recognised by adolescents but not by younger children (Buhrmester 1990; Azmitia et al. 1998; Berndt 2002). Thus, qualitative changes in friendships are related to an age-specific social understanding, which, in turn, will determine the social interaction with peers. Therefore, genetic variation affecting competence in close relationship skills (e.g. intimacy and loyalty) during adolescence (Buhrmester 1990) may not necessarily impact on relationship skills during middle childhood.

Finally, changes in the composition and quality of peer groups and social interactions with age may draw out different genetic propensities (known as gene–environment correlation), which could explain the genetic heterogeneity across development. Evidence suggests that peers are a crucial influence on young people’s outcomes, especially in adolescence when young people have more opportunities to select their own peer groups. The increased genetic stability with age accompanies this increased control in selecting social networks in adolescence compared to earlier in childhood. This interplay between genetic and environmental features may represent a form of social calibration of genetic influences as the young people discover and settle into their personality and peer group behaviour during this developmental stage.

Further investigations are, however, required to explain the observed change in GCTA-heritability in more detail, with, for example, accurate assessments of pubertal status via sex hormone measures.

In this study, we have focussed our subsequent genome-wide screen on the phenotypes with the highest GCTA-heritability (10, 13 and 17 years in ALSPAC). This genome-wide scan did not identify any genetic variation at the genome-wide significant level. However, given the observed genetic heterogeneity during development, which is likely to extend into adolescence, and the increase in GCTA-heritability with progressing age, the lack of genome-wide findings needs to be placed in perspective. First, the population-based follow-up sample (TEDS) was smaller and thus less powerful than the discovery sample (ALSPAC) though in the combined ALSPAC and TEDS sample (e.g. at the age of 10–11 years, *N* = 8,455) there was more than 80 % power to detect genetic variation with MAF of 0.02 explaining as little as 0.5 % of the phenotypic variance at the genome-wide significance level (assuming for simplicity complete LD between marker and causal locus, and a normal phenotype distribution; Genetic power calculator, http://pngu.mgh.harvard.edu/~purcell/gpc/). Second, within TEDS, there was no evidence for measurable common genetic effects contributing to peer problems during early, middle and late childhood as captured by GCTA-*h* ^2^, including the phenotypic measures selected for GWAS follow-up. Finally, assuming that genetic heterogeneity persists throughout development, common genetic signals underlying peer problems in ALSPAC children, especially during later adolescence, may be different to genetic influences contributing to problematic peer behaviour in TEDS children during middle and late childhood.

An exploratory analysis within two ASD samples identified tentative support for two population-based signals (observed in ALSPAC) on chromosome 9p24.2 (rs7873232, at 13 years) and 5p13.1 (rs6451614, at 17 years) within the AGRE but not the ACC sample, assuming the same direction of effect. These findings are consistent with the hypothesis of underlying autism QTL, but also with chance. Closer investigation of these signals in ALSPAC showed that SNP effects vary over time. However, variability in genetic effects during childhood and adolescence is likely to be a common phenomenon in typically developing children. As such, these SNP signals are consistent with our findings from twin analysis and GCTA suggesting genetic heterogeneity during development, while autistic traits are typically characterised by high genetic stability (Holmboe et al. 2014). In addition, there is considerable complexity in behavioural difficulties that may accompany ASD, and recent research pointed out that this may not be sufficiently captured by the peer problem scale of the SDQ (Russell et al. 2013). Thus, overall, our findings provide little evidence to support the hypothesis of peer problems as a broader autistic trait.

In summary, our study showed that peer problems are highly heritable throughout development. Their genetic architecture is, however, complex and involves an increase in measurable common genetic effects during later adolescence as well as genetic heterogeneity.
